# The T Cell Response to *Staphylococcus aureus*

**DOI:** 10.3390/pathogens5010031

**Published:** 2016-03-17

**Authors:** Barbara M. Bröker, Daniel Mrochen, Vincent Péton

**Affiliations:** Department of Immunology, University Medicine Greifswald, Sauerbruchstraße DZ7, 17475 Greifswald, Germany; mrochend@uni-greifswald.de (D.M.); vincent.peton@uni-greifswald.de (V.P.)

**Keywords:** T cell subsets, anti-bacterial defense, adaptive immunity, vaccination

## Abstract

*Staphylococcus aureus* (*S. aureus*) is a dangerous pathogen and a leading cause of both nosocomial and community acquired bacterial infection worldwide. However, on the other hand, we are all exposed to this bacterium, often within the first hours of life, and usually manage to establish equilibrium and coexist with it. What does the adaptive immune system contribute toward lifelong control of *S. aureus*? Will it become possible to raise or enhance protective immune memory by vaccination? While in the past the *S. aureus*-specific antibody response has dominated this discussion, the research community is now coming to appreciate the role that the cellular arm of adaptive immunity, the T cells, plays. There are numerous T cell subsets, each with differing functions, which together have the ability to orchestrate the immune response to *S. aureus* and hence to tip the balance between protection and pathology. This review summarizes the state of the art in this dynamic field of research.

## 1. Introduction

For almost 30 years, the relationship between *Staphylococcus aureus* (*S. aureus*) and T cells has intrigued microbiologists and immunologists alike. In 1987, only three years after the molecular characterization of the T cell receptor, the mode of action of the staphylococcal enterotoxins was elucidated by Bernhard Fleischer and Hubert Schrezenmeier [1]. They showed that these bacterial toxins—the so-called superantigens—are extremely potent T cell activators. To date, 23 superantigen genes have been described in *S. aureus* [2]. While it can only be speculated what advantage *S. aureus* may gain from excessive T cell activation, two conclusions may be drawn: first, T cells must be important in this pathogen-host interaction; and, second, the T cell reaction to *S. aureus* bears pathogenic potential.

*S. aureus* is a notorious pathogen causing a broad range of infections both in and outside hospitals [3,4]. However, persistent colonization of nose and skin is around 1000 times more common than are infections necessitating treatment [5,6,7,8]. Most individuals are continually exposed to *S. aureus* from the first hours after birth and yet manage to establish and maintain equilibrium with the micro-organism for a lifetime [9]. How is this achieved? When does it fail? In the following, our current knowledge of the roles of T cells both in protection and in *S. aureus*-related immune pathology will be discussed. General properties of T cells as well as of innate lymphoid cells (ILCs), their “older siblings”, will be reviewed in the Appendix.

The facultative pathogen *S. aureus* commands an impressive armory of virulence factors, many of which challenge the immune system [10,11,12]. Defense against this “superbug” relies first and foremost on the innate immune system in which professional phagocytes, mainly neutrophils, act in concert with the complement system [13,14,15]. The adaptive immune system, comprising T cells, B cells and antibodies, can facilitate, focus and enhance innate immunity [16].

**Figure 1:**
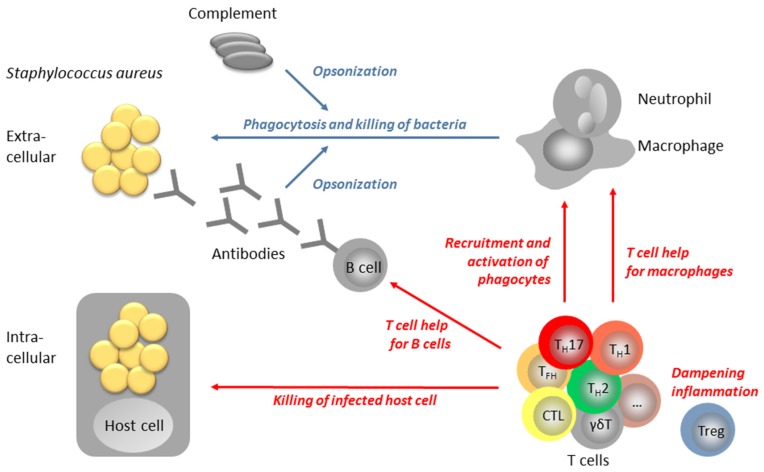
Model of the multifaceted role of T cells in anti-staphylococcal immune defense. The immune defense against *Staphylococcus aureus* crucially depends on the bactericidal activity of phagocytes, especially neutrophils and macrophages. Uptake and bacterial killing is greatly facilitated by opsonization of the bacteria by complement and/or antibodies. T cells can contribute to this process of opsonophagocytosis by: (1) providing help for B cells; (2) promoting the bactericidal potential of macrophages; and (3) recruiting phagocytes from the bone marrow to the site of infection. Once *S. aureus* has invaded host cells to persist in intracellular niches, cytotoxic T cells would be required to kill the infected cell, releasing *S. aureus* for a second round of opsonophagocytosis. Tregs have a vital function in dampening inflammatory processes and restoring homeostasis after resolution of the infection. CTL: Cytotoxic T lymphocyte; T_FH_: T follicular helper cell; T_H_: T helper cell; Treg: regulatory T cell.

How can T cells contribute to antibacterial immunity? Extracellular *S. aureus*, bacteria are engulfed and destroyed by phagocytes and this process is greatly facilitated by the binding of specific antibodies to the microbial surface. Such antibodies opsonize *S. aureus* for phagocytosis via complement activation and immunoglobulin-Fc receptors on phagocytes [15]. In this process of opsonophagocytosis, T lymphocytes have a dual function: On the one hand, they are important for the generation of opsonizing antibodies, because T cell help is required for antibody affinity maturation as well as for class switch [17]. On the other hand, T cells promote phagocytosis by recruiting neutrophils and macrophages from the bone marrow to the site of infection [18]. However, *S. aureus* is not exclusively an extracellular bacterium but can also adapt to the intracellular milieu, which enables it to persist and even divide inside host cells [19]. As long as the bacteria reside inside macrophage phagosomes their elimination is promoted by T cell cytokines, most prominently by IFN-γ [18]. However, once the bacteria escape from the phagosome into the cytoplasm, they are beyond the reach of T helper cells (T_H_). The infected cell must now be lysed by cytotoxic T cells (CTLs) or by natural killer cells (NK) [20] so as to release the bacteria and make them accessible to a new round of phagocytosis and killing. Finally, T cells are essential for (re)establishing immune homeostasis by dampening inflammatory processes. Regulatory T cells (Tregs) are specialized in this vital immune function [18]. Figure 1 shows a model of the potential roles of T cells in the anti-*S. aureus* defense network.

## 2. The role of T Cells in *S. aureus* Control

There is now general agreement that T cells are important for protection from *S. aureus* infection [21,22]. This is based on observations in humans, livestock and experimental animals. Many groups have contributed evidence for the importance of T cell-derived IL-17 as well as IFN-γ.

### 2.1. Evidence from Mouse Models

In experimental mouse models multiple factors influence the susceptibility to *S. aureus* infection, the decisive T effector cell type as well as the protective cytokines: (1) the mouse strain; (2) the *S. aureus* strain; and (3) the type and duration of the infection. BALB/c mice, for example, rapidly succumb to blood stream infection with *S. aureus*, while C57BL6 mice are more resistant [23]. The opposite is true in skin and soft tissue infection [24,25]. The multifaceted role of T cells in the interplay between pathogen and host may be illustrated by a model of systemic *S. aureus* infection in C57BL6 mice, where it differed between the acute and chronic disease phase. In early disease, animals deficient in T cells, B cells and NK cells had no apparent defect in *S. aureus* clearance; T cells appeared to be dispensable [23]. However, during chronic infection—mice were monitored for 60 days—T cells made an important contribution to *S. aureus* control, even though the animals were not able to clear the infection completely [26]. Remarkably, while the T cells proliferated vigorously when exposed to the bacteria *ex vivo* during acute infection, this response was completely abolished in the chronic phase. Not only was the specific anti-*S. aureus* T cell response affected, but T cell reactivity generally was blunted [26]. The profound T cell suppression in chronic *S. aureus* infection was attributed to myeloid-derived suppressor cells (MDSCs) with a minor contribution from Tregs [27].

Following experimental colonization, C57BL6 mice spontaneously cleared *S. aureus* from the nose within 14 d. This was dependent on T cells, IL-17 and neutrophil influx but not on antibody production or IL-23 [28].

In skin and soft tissue infection, IL-17 had a crucial role in bacterial clearance, limiting lesion size. Cytokines of the IL-17 family may be generated by different cell types [29,30,31,32]. In a seminal study the group of Ishigame identified CD4^+^ T_H_17 cells as the main producers of IL-17A, whereas IL-17F was released by a broader range of cells [33]. Cho and coworkers reported γδ T cells to be the main producers of IL-17 in skin and soft tissue infection [34]. Similarly, in a murine model of wound infection, γδ T cells infiltrated the site of infection within three days, and many of them secreted IL-17, which reduced the local bacterial load [35]. In line with this, genetic deficiency in γδ T cells increased the bacterial burden in skin infection [36]. With repeated cutaneous infection the *S. aureus*-induced lesions became smaller in BALB/c but not in C57BL6 mice. Both T_H_17 cells and antibodies were induced and each provided some degree of protection upon adoptive transfer [25].

Similarly, in peritonitis, IL-17 producing γδ T cells acquired memory behavior, reacting more rapidly upon *S. aureus* re-challenge [37].

For survival of blood stream infection, in contrast, IFN-γ-competent CD4^+^ T cells were essential [38]. In accordance with this, the time course of disease did not differ between WT and IL-17-KO mice in a lethal intravenous (i.v.) infection model (C57BL6) [39]. When mice with severe combined immune deficiency (SCID), who are genetically deficient of adaptive immune cells, were reconstituted with human immune cells, the activation of the γδ T cell pool greatly improved the control of systemic *S. aureus* infection. This was correlated with the production of human IFN-γ. IL-17 measurements were not reported in this early study [40].

*S. aureus* pneumonia, is special in that the inflammatory mechanisms of anti-bacterial defense easily cause life threatening organ damage by interfering with gas exchange in the lungs. This may explain the ambiguous role of T cells documented in murine pneumonia models (all using C57BL6 mice). Following infection, *S. aureus* quickly activated conventional T lymphocytes in the lungs as well as in the draining lymph nodes, and this could be attributed partly to superantigen action [41]. Another group observed rapid accumulation of γδ T cells in the lungs, and γδ T cell-deficient animals showed delayed IL-17 production, neutrophil influx and bacterial clearance, implying a protective role for this T cell subset in *S. aureus* pneumonia. On the flip side of the coin, tissue damage was more severe in γδ T cell-competent animals, and these animals were sicker. Nevertheless, in this model survival was similar both in γδ T cell competent and in deficient animals [42]. Another study documented important roles for IL-17 and IL-22 in protection from *S. aureus* pneumonia. IL-17 was produced by both CD4^+^ T_H_17 cells and γδ T cells. Co-infection with influenza increased bacterial burden and death. This was correlated with impairment of the IL-17 (and IL-22) response, while rescuing the IL-17-production by IL-23 application led to increased survival of the animals [43]. While this makes the case for a protective role of T cells in *S. aureus* pneumonia, the group of Parker reported a detrimental role of T cells since all CD4^+^ T cell-deficient mice survived, whereas all T cell competent animals died. The improved survival of the T cell-depleted animals was associated with reduced inflammation, e.g., abolishment of IL-17 release into the bronchoalveolar lavage fluid. Despite the dampened inflammation, bacterial clearance was more efficient in T cell-deficient animals [41].

#### Vaccination Studies in Mice

Does the encounter with *S. aureus* or its components induce immune memory, and if so, does this confer clinical protection? Two strategies have been followed to answer this question: First, immunization with *S. aureus* components or whole inactivated *S. aureus* cells, and second, immunization with live *S. aureus*. The latter can be considered both as a vaccine approach and as a model of recurrent infection. Numerous vaccine studies in mice have shown clinical protection, which, while usually not sterilizing, clearly mitigated the disease course and reduced the bacterial load during challenge infection [44]. T cells have been shown to be essential for vaccine protection; depending on the model used either IL-17 or IFN-γ was the crucial protective cytokine.

In *S. aureus* bloodstream infection, immunization with ClfA in alum reduced the bacterial burden and improved survival. Interleukin-17 was required for protection. In contrast, anti-ClfA antibodies, which were induced in abundance, did not confer protection upon transfer to naïve animals [39]. IL-17-producing T lymphocytes were also essential for protection against *S. aureus* blood stream infection following vaccination with Als3p (in alum), an antigen derived from *Candida albicans* conferring cross-protection against *S. aureus* [38]. Similar results were obtained with the *S. aureus* iron scavenger IsdB as a vaccine antigen. In this case, specific antibodies, which were elicited by the vaccine in large amounts, were also protective but only in T cell-competent animals. The effect of passive vaccination with a monoclonal antibody targeting IsdB depended on the presence of CD4^+^ T cells as well [45]. Passive vaccination by surface-binding antibodies such as the anti-IsdB monoclonal antibodies is assumed to act by opsonizing *S. aureus* for phagocytosis. In this setting, the crucial role of IL-17-producing T cells can be explained by the fact that phagocyte recruitment and activation is strongly amplified by the action of this cytokine. While the protective action of antibodies depended on functional T cells, the opposite was not true: Even in the absence of neutralizing antibodies, protection from systemic *S. aureus* infection has been achieved after nasal vaccination with *S. aureus* ClfA coated on nanoparticles [46]. Moreover, in combination with a T_H_1 polarizing adjuvant the vaccine antigen ClfA conferred clinical protection in an *S. aureus* peritonitis model [47]. Finally, a peptide construct mimicking *S. aureus* peptidoglycan—followed by a boost with inactivated *S. aureus* cells—provided partial protection from an i.v. challenge infection. This was associated with increased numbers of T_H_1 and T_H_17 cells in the spleen [48].

Repeated *S. aureus* infection—or vaccination with live bacteria—conferred clinical protection as well. In skin and soft tissue infection, both T_H_17 cells and antibodies contributed to the observed reduction of lesion size in *S. aureus*-experienced animals [25]. The *S. aureus* bacterial load was also significantly reduced when a high dose challenge infection was preceded by repeated peritoneal infection with a low bacterial dose. In this case protection was associated with strongly increased production of IFN-γ by peritoneal CD4^+^ and CD8^+^ T cells. Remarkably, *in vitro*-expanded *S. aureus*-specific T_H_1 but not T_H_17 cells from *S. aureus*-primed animals transferred clinical protection to naïve mice. The bacterial burden in the recipient animals was reduced about 100-fold, a remarkably large effect [47]. Interestingly, in the same infection model, IL-17-producing γδ T cells were more effective at the initial stage of infection [49]. Hence disease kinetics adds a further layer of complexity to the effects of T cells in *S. aureus*-host interaction.

### 2.2. Evidence from Livestock

In livestock, especially in cattle, *S. aureus* is one of the leading infective agents in acute and chronic mastitis causing substantial economic losses worldwide [50,51,52]. Estimated costs range from US$ 38–130 per dairy cow and year [52,53]. The decrease in milk production and the need to dispose of the milk of infected animals are the main cost factors [54]. Therefore, most of the few studies addressing the interplay between *S. aureus* and T cells in livestock are focused on bovine mastitis.

In comparison with healthy cows, animals suffering from staphylococcal mastitis had significantly elevated numbers of γδ T cells in the blood, whereas both αβ T cells and γδ T cells were increased in the milk [55]. The increase of αβ T cells in the milk was mainly due to CD4+ T cells, and several groups reported increased CD4+:CD8+ ratios in cattle with mastitis [55,56,57]. However, there are also contrasting reports of lowered CD4+:CD8+ ratios in *S. aureus* mastitis [58,59,60]. Cows having substantially more CD4+ T cells than CD8+ T cells in their mammary gland secretions appear to be more mastitis-resistant suggesting that CD4+ T cells are protective [58]. The role of γδ T cells, which appear to be preferentially recruited to the mammary gland during infection, remains to be elucidated [55].

#### Vaccination Studies in Cattle

Vaccination could be a promising way to prevent intramammary infections. Many vaccine types have been developed, some are commercially available (reviewed in [61,62,63,64,65]). Lee *et al.* described the use of heat killed *S. aureus* strains producing the main capsular antigens found in mastitis strains [66]. Capsular polysaccharides alone have also been tested as potential vaccine antigens [67], as have been toxoids such as α-toxin and hemolysin β, which are regularly produced by bovine *S. aureus* strains [66,68]. Despite decreasing mastitis occurrence and accelerating remission, none of them proved to be fully able to prevent new infections [69,70,71,72]. Vaccines based on live attenuated *S. aureus* strains have been tested with promising results in terms of antibody generation [73,74,75]. The T cell response, however, has not been studied in the described vaccination trials.

Lawrence and coworkers immunized calves with heat-killed *S. aureus* and tested 43 recombinant *S. aureus* proteins for eliciting a recall T cell response. The screen identified 13 T cell antigens that were able to induce strong proliferation in CD4+ T cells and may therefore represent novel vaccine targets [76]. In order to activate CD8+ T cells as well, Pujol *et al.* used a soluble trimeric form of bovine CD40L as an adjuvant in *S. aureus* vaccination in heifers. Three animals were vaccinated on one side with non-adjuvanted and on the other with adjuvanted heat killed *S. aureus*. The bovine CD40L enhanced the number of antigen-responsive CD4+ and CD8+ T cells in the draining lymph node as well as their reactivity [77]. Challenge infections were not performed in these studies.

### 2.3. Evidence from Humans

In humans, equilibrium with *S. aureus* is usually maintained for many years or even decades [78]. Epidemiological evidence for a role of T cells comes from observations in patients suffering from inborn or acquired T cell deficiencies. These provide indirect evidence for a protective function of T cells. Patients with genetic defects in T_H_17 cell development and function are highly susceptible to *S. aureus* infection [79,80,81,82,83,84]. *S. aureus* is also among the major pathogens affecting HIV patients, once the virus has strongly reduced CD4^+^ T cell counts and functions [85,86]. In a Canadian study, HIV infection increased the relative risk of *S. aureus* invasion by a factor of 17 [87], and in Denmark it was discovered that HIV patients with *S. aureus* blood stream infection are also more prone to develop a secondary disease episode than HIV-negative persons [88].

T cell involvement in *S. aureus* interaction with its human host is also implied by the strong antibody response targeting a broad repertoire of *S. aureus* antigens in health as well as infection [89,90,91]. The demonstration of a strain-specific component in an individual’s anti-*S. aureus* antibody profile makes a strong case for the notion that the antibodies are generated in an antigen-driven process rather than being produced due to polyclonal B cell activation. [92,93]. The generation of antigen-specific, high affinity class switched antibodies, however, requires the help of T cells specific for the same antigen [94,95].

Using inactivated *S. aureus* cells to stimulate isolated T cell subpopulations, the group of Zielinski found reactive T cells predominantly in the T_H_1 and T_H_17 subsets, but some T_H_2 cells could also be stimulated by the microbe [96]. Other groups also observed proliferation of human T cells when blood cells of healthy donors were stimulated with inactivated *S. aureus* cells [47,97]. Brown and coworkers reported that IFN-γ was the dominant cytokine released into the cell culture [47]. In another approach, Zielinski and coworkers primed human blood cells with inactivated *S. aureus* cells *in vitro*. This resulted in the expansion of T_H_17 cells, which upon re-stimulation acquired the ability to produce IL-10 in addition to IL-17 [98]. A T_H_1/T_H_17 profile was also most common in T cell clones responding to extracellular *S. aureus* antigens [97]. To study the magnitude of the human *S. aureus*-specific T cell pool, Kolata and coworkers challenged human peripheral blood T cells from healthy adults with a number of recombinant *S. aureus* proteins selected from the extracellular bacterial proteome. The observed frequencies of the reacting T cells corresponded to a memory response in most cases. Remarkably, there was a 35-fold difference between high and low responders, probably reflecting the individuals’ history of (invasive) encounters with *S. aureus*. The group estimated that on average more than 3% of the peripheral T cells were specific for *S. aureus*, underlining that maintaining an equilibrium with *S. aureus* places a heavy cost on the immune system [97].

In a seminal clinical study, Brown and coworkers examined patients with blood stream infection and found increased IFN-γ serum concentrations in *S. aureus* but not *E. coli* infection. The patients’ CD4^+^ and CD8^+^ T cells proliferated *ex vivo* in response to *S. aureus* in an antigen-specific manner, and the CD4^+^ T cells produced IFN-γ. Under the same conditions, the number of IL-17-secreting cells was low [47].

To date, the analysis of the natural human T cell response to *S. aureus* has been focused on inflammatory T helper cells of the T_H_1/T_H_17 type. However, type 2 cytokines may also be released by *S. aureus*-specific T cells [97,98,99]. Importantly, anti-inflammatory effects such as those exerted by Tregs have not been studied in humans. They merit attention, because Tregs may act as a double-edged sword in infection, limiting organ damage due to inflammation at the price of interfering with bacterial clearance thereby promoting chronification [100,101,102].

The search for the molecular targets of *S. aureus*-responsive human T cells is still in its infancy. The most comprehensive study has been conducted by the group of Kolata who enumerated *S. aureus*-specific T cells in healthy donors. Taking the human antibody response as a lead for antigen selection, they found T cells reacting to α-toxin (Hla), the phosphodiesterase GlpQ, the phospholipase Plc, the lipases Geh and Lip, the proteases SplC and D, as well as the iron scavenger IsdB. Remarkably, the T cell response to the IsdB was very low in most tested individuals [97] suggesting that vaccination with this antigen will greatly increase the specific T cell response in humans [103] as has been shown in mouse models [45]. Similar observations were reported for ClfA in healthy adults [47]. Alpha-toxin has been shown to induce a T_H_1 response as well as IL-17 production by T cells in humans [104,105]. In another study, staphylokinase (Sak)-specific T cells were measured in healthy human adults. The intensity of the natural T cell reaction varied strongly and generally increased with age [106]. It is not only proteins that can be recognized by T cells, for wall teichoic acids have also been demonstrated to elicit a T cell response both in mice and humans [97,107]. Extending the analysis to unconventional T cells and ILCs may uncover further *S. aureus* targets in the future.

#### Vaccination Studies in Humans

T cell responses have not yet been analyzed in human vaccine trials—with one recent exception [108]—since the vaccine approaches have targeted the antibody response aiming at maximizing opsonization capacity [109]. Unfortunately, these efforts have not met with success and did not result in significant protection [110,111,112,113,114,115]. Moreover, a large clinical trial raised a safety concern. When patients awaiting cardiothoracic surgery were vaccinated with the non-adjuvanted *S. aureus* protein IsdB, *S. aureus* infection occurred with similar frequency in vaccinated and non-vaccinated patients. Mortality, however, was around fivefold higher in the vaccinated persons [103]. Retrospectively it was shown that vaccinated patients who succumbed to *S. aureus* infection had undetectable serum levels of IL-2—and in most cases IL-17—at the time of vaccination, infection and during the course of the disease. [116]. Apparently, the patients’ pro-inflammatory T cells were unable to react to vaccination or infection. It is tempting to speculate that in these patients the vaccine had enhanced pre-existing mechanisms of active suppression, possibly by boosting specific Tregs.

The use of recombinant Sak for fibrinolytic therapy in myocardial infarction provides a window of opportunity for the study of *S. aureus* specific T cell-reactions in humans. Intravenous exposure to Sak increased the specific T cell response four weeks later. In one individual the time course of the T cell response was monitored, showing that the increase was maximal after four weeks but had not returned to baseline at 44 weeks, just as one would expect of a T cell memory response [106]. The influence of the elicited *S. aureus*-specific effector and memory T cells on the course of subsequent *S. aureus* infections was not addressed in this study. In a recent phase I clinical trial of an *S. aureus* vaccine composed of four antigens specific memory T cells were induced at low levels, whereas the vaccine elicited a robust antibody response. The reasons for this discrepancy are as yet unknown [108]. Nevertheless, the findings indicate that T cells respond to vaccination with *S. aureus* antigens. The quality of the T cell response elicited by different *S. aureus* antigens and its effects on subsequent infection remain to be investigated.

### 2.4. A Special Case: S. aureus Persisting Inside Host Cells

It is now well established that, besides its typical extracellular life-style, *S. aureus* is able to persist inside a number of cell types: epithelial and endothelial cells, keratinocytes, osteoclasts, as well as in professional phagocytes such as macrophages and neutrophils [19,117,118]. Intracellular *S. aureus* could be an endogenous reservoir for re-colonization and/or -infection, and the intracellular life-style of the micro-organism has been implied in the pathogenesis of chronic and difficult to treat *S. aureus* disease such as osteomyelitis [117]. It appears that the bacteria may even use granulocytes as Trojan horses facilitating metastatic infection and abscess formation, which may complicate *S. aureus* blood stream invasion [119]. Long-term intracellular persistence is associated with profound bacterial adaptation processes as has been shown both in cell culture and in infection models. The bacteria assume a dormant state known as small colony variant (SCV), which is reversible as soon as they conquer a more permissive niche or are taken into culture [120,121,122,123]. Host cells react to *S. aureus* invasion with an inflammatory response [122,124]. It is presently not known how T cells react to intracellular persisting *S. aureus* and whether they have a role in controlling the bacteria that have invaded this protected habitat. The cytokine IFN-γ is a potent activator of monocytes, and pre-treatment of host monocytes with IFN-γ dramatically increased their potential for killing *S. aureus* [125,126]. IFN-γ may be produced by a number of immune cells, namely αβ T cells, both CD4^+^ and CD8^+^, γδ T cells, NK cells as well as by ILC1. Thus all of these are of interest in this context. Moreover, the potential role of cytotoxic cells, CTLs and NK cells, should be addressed in the future.

### 2.5. The T Cell Response to S. aureus May Cause Harm

While defects in the adaptive immune response had been the main concern of researchers for a long time, the *S. aureus* research community is now weighing the possibility of immune pathology, such as hyper-inflammation or allergies elicited by *S. aureus*. T cells can contribute to such noxious inflammatory reactions, where damage of the host tissue appears to be out of proportion to the beneficial anti-bacterial effects. The scenario of T cell mediated hyper-inflammation can be dramatic as has been illustrated in a mouse model of viral infection. Priming of CD4^+^ T cells in the absence of a neutralizing antibody response caused a detrimental cytokine storm [127]. Enhancing the anti-*S. aureus* T cell response by vaccination may, therefore, carry the risk of promoting damaging hyper-inflammation.

Neutrophils are essential for *S. aureus* clearance, and T cells foster their action, e.g., by the release of IL-17, which recruits neutrophils to the site of infection, and of IFN-γ, which extends their life span. This amplifies inflammation contributing to bacterial clearance but at the same time increasing the danger of organ damage. More than a decade ago, it was reported that γδ T cells accumulating in *S. aureus* skin infection reduced the bacterial burden but increased the local inflammation [36]. T cells were critically involved in abscess formation induced by staphylococcal wall teichoic acids [107], and in a model of repeated skin infection, neutralization of the T_H_1 signature cytokine IFN-γ reduced lesion size [25]. The recent findings of McLoughlin’s group of a T_H_1-dominated immune response in human severe systemic *S. aureus* infection may point into the same direction [47]. As discussed above, the lungs are particularly sensitive to inflammation-induced organ damage and T cells contribute to this life-threatening condition [41,42]. Finally, it has been shown that, counterintuitively, neutrophil influx may expand a niche for those *S. aureus* strains that are able to persist inside the phagocytes [119,128].

*S. aureus* has also been implicated in allergies including intrinsic asthma and atopic dermatitis [129,130,131]. Allergic or type 2 inflammation is characterized by the generation of allergen-specific IgE, and T_H_2 cells have a crucial role in this form of immune pathology [132]. *S. aureus* is capable of exacerbating allergic inflammation by releasing factors that either favor the differentiation of naïve T cells into T_H_2 cells or activate pre-existing T_H_2 cells. Proteases as well as the pore-forming α-toxin disrupt the epithelial barrier facilitating allergen entry [133,134,135], while δ-toxin triggers mast cell degranulation, a central allergic effector mechanism [136]. *S. aureus* superantigens (see below), which potently stimulate T cells irrespective of their antigen specificity or function, can exacerbate allergic inflammation [129,137,138]. Moreover, our group has recently discovered that *S. aureus* releases allergens, namely proteases that are capable: (1) of eliciting a T_H_2 response in healthy adults; and (2) of driving allergic airway inflammation in mice [99].

### 2.6. How S. aureus Manipulates T Cells

*S. aureus* targets T cells mainly with two classes of virulence factors, pore-forming toxins and superantigens. In addition, the “superbug” has different means of modulating the quality of the anti-staphylococcal T cell response. All of this is circumstantial evidence for the importance of T cells in keeping *S. aureus* at bay.

T cells can be lysed by two extracellular *S. aureus* toxins: α-toxin is released in variable amounts by at least 95% of clinical *S. aureus* isolates. Upon binding to ADAM10 on the host cell surface [139], α-toxin forms heptameric pores, thereby destroying its target cells, among which are T cells [133]. Leukocidin LukDE is a two component toxin which lyses its targets after binding to the chemokine receptor CCR5, which is present on human T cells as well as on myeloid cells [140].

Due to their remarkable properties, superantigens have been the paradigm of *S. aureus* -T cell interaction for decades, and much is known about their action. The term superantigen was coined by Marrack and Kappler, because these toxins are extremely potent T cell activating agents [1,141]. The pan-genome of *S. aureus* contains 23 superantigen genes, encoding enterotoxins (SEs) and enterotoxin-like proteins (SEls) as well as toxic shock syndrome toxin-1 (TSST-1), most of which are located on pathogenicity islands or mobile genetic elements (reviewed in [2,142,143]). SEs but not SEls or TSST-1 have been shown to possess emetic properties, which is due to serotonin release in the intestine, a mechanism distinct from the superantigenicity of the toxins [144]. Around 80% of *S. aureus* strains, both commensal and pathogenic isolates, harbor superantigen genes, on average five to six [145,146,147,148,149], which are released by the bacteria either during exponential or post-exponential growth [150].

Superantigens are a common cause of food poisoning as well as the causative agents of toxic shock syndrome, a rare but very dramatic condition [142]. The potent T cell stimulation ability of superantigens is explained by the fact that the toxins bypass the need for antigen processing and presentation by directly bridging αβ T cell receptors with conserved structures on MHC-II molecules. A given superantigen targets all αβ T cell receptors that use particular Vβ elements, up to 20% of the whole T cell repertoire. The simultaneous activation of such large T cell numbers results in a cytokine storm manifesting itself as life-threatening toxic shock syndrome [2,142,151,152].

When studying the T cell response to *S. aureus* in cell culture, superantigen action may override T cell activation by conventional antigens, because these toxins can be released by the bacteria in large amounts, act at minute concentrations and activate large fractions of T cells. When addressing the antigen-specific T cell response to *S. aureus*, care should be taken to avoid artifacts caused by contaminating superantigens. Since these are robust molecules that cannot not reliably be inactivated by heat treatment or proteases, this is best achieved using recombinant *S. aureus* proteins to probe the anti-staphylococcal T cell repertoire [97].

The possible benefits conferred to *S. aureus* by superantigens remain a matter of discussion. During the cytokine storm of toxic shock syndrome T cells become refractory to antigen-mediated activation. They are then unable to help B cells in mounting an *S. aureus*-specific antibody response. In this scenario, superantigens act as a “smoke screen” hiding *S. aureus* from the adaptive immune system [142,153]. Moreover, during fulminant systemic inflammation, the high concentrations of the cytokine TNF in the blood interfere with phagocyte recruitment to the site of infection [142,154]. This may be due to “atopic” activation and sequestering of the cells inside the blood vessels. However, despite the fact that everyone is exposed to *S. aureus* in everyday life, toxic shock syndrome is a very rare condition with a yearly incidence between 3 and 7 per million inhabitants in the US between 2000 and 2006 [155]. Moreover, during harmless encounters with the bacteria such as colonization or during mild infection, superantigens themselves act as highly immunogenic conventional antigens eliciting a protective specific antibody response, which neutralizes their toxic effects [92,93,156]. This is at odds with a major superantigen function as “smoke screens”. An alternative idea is, therefore, that superantigens may promote *S. aureus* colonization and/or minor infections thereby facilitating bacterial persistence and transmission [143,157,158].

Besides activating or killing T cells by the two types of toxins, *S. aureus* can influence the quality of the T cell response, counteracting the development of an anti-bacterial T_H_1/T_H_17 profile. This may be the evolutionary advantage conferred to the microorganism by its multi-pronged pro-allergenic activity, which has been described above (Section 2.5). Peptidoglycans, components of the *S. aureus* cell wall, have been shown to elicit the release of the anti-inflammatory cytokine IL-10, thereby dampening the T_H_1/T_H_17 response [100,159]. Finally, phenol-soluble modulins, amphiphilic peptides that are cytolytic at high concentrations, favor the differentiation of Tregs [160], which inhibit inflammatory T cell responses.

## 3. Conclusions and Future Directions of Research

The development of an *S. aureus* vaccine is an ambitious goal as underlined by the many futile attempts in the past. Understanding the T cell response to the bacteria may be key for success, because successful vaccination relies on immune memory, a core competence of the adaptive immune system where T lymphocytes have a central role. It will, therefore, be of prime importance to address the following points: What T cell and innate lymphoid cell subsets react to *S. aureus in vivo*? What antigens do they recognize? How does *S. aureus* manipulate the strength and quality of the T cell response?

Ideally, a vaccine induces an immune memory response of appropriate quality targeted at the right antigens. But what is the appropriate quality, what are the right antigens? In order to answer these questions, we urgently need to identify correlates of protection from *S. aureus* disease, many of which may be expected in the T cell compartment. To identify and employ such biomarkers, simple tests need to be developed that are suitable for measuring the anti-*S. aureus* T cell response “at the bedside”, namely at high throughput and with small amounts of blood.

There is now broad consensus that memory T cells make an essential contribution toward *S. aureus* control. A number of different *S. aureus* antigens could serve to elicit *S. aureus*-reactive T_H_1 or T_H_17 cells that will recruit neutrophils to the focus of infection, so long as these antigens are conserved in *S. aureus* and are generated in sufficient amounts upon bacterial invasion. Similarly, if T cells were required for dealing with intracellular bacteria, *S. aureus* proteins that are abundant in the invasive microorganisms and that can readily be processed and presented to the T cells by the infected host cells should be selected.

On the other hand, when targeting T cells by vaccination, adverse effects, such as those caused by excessive reaction of the primed T cells to *S. aureus* infection, have to be avoided as well as the preferential generation of Tregs. As a prerequisite kinetics and plasticity of the T cell reaction to *S. aureus* have to be explored. Fine-tuning the quality of the immune response elicited by a vaccine may be achieved by careful selection of adjuvants, while the adjuvant properties of *S. aureus* components themselves should not be ignored, especially when designing non-adjuvanted vaccines.

## Abbreviations

ClfA	clumping factor A
CTL	cytotoxic T lymphocyte
DAMP	Damage-associated molecular pattern
DC	dendritic cell
ILC	innate lymphoid cell
i.v.	intravenous
MAIT	mucosa associated invariant T cell
MAMP	microbe-associated molecular pattern
MDSC	Myeloid derived suppressor cells
MHC	major histocompatibility complex
NK cell	natural killer cell
Sak	staphylokinase
SCID	severe combined immune deficiency
SCV	Small colony variant
SE	Staphylococcal enterotoxin
SEl	Staphylococcal enterotoxin-like
TCR	T cell receptor
T_FH_	T follicular helper cell
T_H_	T helper cell
Treg	regulatory T cell
TSST-1	Toxic shock syndrome toxin-1

## Appendix

### A1. The World of T Cells and Innate Lymphocytes

T-cells are equipped with antigen-specific clonotypic T cell receptors (TCR), which are key to their activation. In around 95% of human T cells, the TCR is composed of covalently linked α and β chains, in the remainder of γ and δ chains. While the TCRs on a single T cell are molecularly identical (a number of T cells expresses two TCR α chains, which may each combine with the singular TCR β chain resulting in two different TCRs), there is an impressive diversity of TCRs at the level of the T cell population. This is due to a unique process that T cells share only with the antibody-producing B cells: In each developing T cell the TCR-encoding genes are assembled from variable genetic elements by DNA recombination. This results in an extraordinary diversity of TCRs, in particular of those peptide loops which contact the antigen complex. This is at the basis of the exquisite antigen specificity of individual T cells as well as the T cell response as a whole [161].

Normal cells in the body display on their surface peptides, which represent the intracellular proteome. These peptides are displayed in complex with host cell molecules encoded in the major histocompatibility gene complex (MHC). Through the processes of central and peripheral tolerance T cells expressing TCRs specific for self peptide-MHC complexes are eliminated, silenced or they develop into Tregs with anti-inflammatory function. The remaining T cells now use their TCR to scan the surfaces of host cells for those which display “non self” antigens, e.g., peptides derived from infectious agents, complexed with host cell MHC molecules.

However, the down side of the extraordinary diversity of TCRs is the very low number of naïve T cells (naïve T cells are mature T cells which have not yet encountered their specific antigen) specific for a given antigen (=peptide-MHC complex). Specific T cell precursor frequencies are estimated to be in the range of 5 to 200 per million T cells in the case of protein antigens [162,163]. For a powerful immune response the few antigen-specific T cells have to undergo many rounds of division greatly expanding the number of antigen-reactive cells. Following immunization with vaccinia virus, for example, antigen-specific T cell numbers increased by a factor of 10^4^–10^5^, such that at the peak of the response, two weeks after vaccination, 3%–14% of the peripheral blood CD8^+^ T cells were specific for the virus [164].

#### A1.1. Signals 1, 2, 3—The Rules of the T Cell Game

First and foremost, T cell activation requires the engagement of their master switch, the antigen-specific TCR. This results in what is known as **signal one**. In most αβ T cells, the TCR recognizes a complex of an antigenic peptide with an MHC I or MHC II molecule, which is presented on a host cell surface. The MHC locus is highly polymorphic at the population level, and T cell activation requires that the antigenic peptide be complexed with a specific MHC allele. Hence, antigen-recognition by the αβ TCR is MHC restricted. The peptides are generated by the antigen-presenting cell from proteins by proteolytic processing and then loaded onto MHC-molecules for presentation [165]. However, signal one is not sufficient for the activation of naïve T cells; rather, a second signal is required in addition. **Signal two** consists of co-stimulation via CD28 on the T cell membrane and can only be provided by professional antigen-presenting cells that, in the case of naïve T cells, are mainly dendritic cells (DC). DCs upregulate expression of the CD28 ligands, CD80 and CD86, only upon perception of danger signals with their innate pattern recognition receptors such as, e.g., the toll-like receptors. The microbial or host cell molecules able to trigger these innate pattern recognition receptors are known as microbe-associated molecular patterns (MAMP) or damage-associated molecular patterns (DAMP), respectively. Thus, linking adaptive to innate immunity, signal two serves as a safeguard against untimely T cell activation. Naïve T cells that are fully activated by signals one and two proliferate and differentiate into effector T cells. Numerous effector T cell subpopulations can be distinguished, which exert specialized functions determining the quality of the immune response (summarized in Table A1). T cell differentiation is guided by **signal three** comprised of micro-environmental cues such as cytokines elaborated by the DCs [166,167]. Signal three, therefore, provides another link between innate immunity and T cells. Depending on their differentiation into helper T cell subpopulations, such as T_H_1, T_H_2, T_H_17, follicular T helper (T_FH_) cells or Treg cells, antigen-experienced CD4^+^ T cells shape the Ig (sub)class composition, support the formation of memory B cells, activate macrophages and/or enforce recruitment and activation of neutrophils [17,18,94,166,168]. CD8^+^ T cells can acquire cytolytic ability and differentiate into CTLs [169].

During resolution of an immune response, the previously expanded T cell clones contract and most antigen-specific T cells die. A minority of the antigen-experienced T cells, however, survive as memory T cells, among which three main types are distinguished: central memory T cells (T_CM_), effector memory T cells (T_EM_) and tissue resident memory T cells (T_RM_). There is evidence that follicular T helper cells can generate memory as well [170]. Upon further encounters with the same antigen, the antigen-experienced T cells generate a memory response, which differs from the primary response in several ways: There are now more antigen-specific T cells, and these react faster than naïve T cells. Moreover, the cells recall and exert the specialized effector functions they acquired during the primary encounter with the antigen thereby defining the quality of memory responses [166].

**Table A1:** Differentiation and functions of T cell subsets.

	Inducing Cytokines	Lineage-Specific Transcription Factor	Control of …	Secreted Cytokines	Main Functions
**T_H_1**	IL-12 IFN-γ	Tbet	Intracellular vesicles	IFN-γ IL-2	Activate macrophages Help CD8^+^ T cells
**T_H_2**	IL-4	Gata3	Extracellular space	IL-4, IL-5, IL-9, IL-13	Recruit eosinophils Orchestrate type 2 inflammation
**T_H_17**	TGF-β IL1-β, IL-6, IL-21, IL-23	RORγT	Extracellular space	IL-17 IL-6	Enhance neutrophil response Help mucosal B cells (IgA)
**T_FH_**	IL-6, IL-21 TGF-β	Bcl6	Extracellular space	IL-21 and others	Help B cells (antibody class switch to IgG, IgA and IgE; antibody affinity maturation)
**Treg**	TGF-β	Foxp3		TGF-β, IL-10	Suppress T cell responses Help mucosal B cells (IgA)
**CTL**			Cytoplasm	IL-2, IFN-γ	Kill infected cells

CTL: Cytotoxic T lymphocyte; T_FH_: T follicular helper cell; T_H_: T helper cell; Treg: regulatory T cell.

#### A1.2. Unconventional T Cells

In addition to the majority of αβ T cells reacting to protein antigens, T cell populations have been discovered whose TCRs are able to recognize non-protein intermediates of microbial metabolism, such as glycolipids [171]. Since these key T cell activating metabolic components are shared by large groups of microorganisms, their function in microbe-host interaction is similar to that of MAMPs, which are sensed by the innate immune pattern recognition receptors. In contrast to peptide epitopes, the microbial metabolic compounds bind to conserved invariant MHC-like molecules on host cells. The corresponding TCR repertoire is also conserved, is near germline and is much narrower than that of conventional αβ T cells. Our knowledge about such “unconventional” T cell populations has increased greatly during recent years but much remains to be discovered. Remarkably, while bacterial compounds are prominent triggers of unconventional T cells, many of these cells display a “memory” phenotype already during fetal development, though obviously in the absence of bacterial stimuli. The reasons for this are not understood, but the feature is of consequence for host-pathogen interaction: Upon primary encounter with their bacterial ligands after birth, unconventional T cells mount a very rapid memory-type immune response thereby contributing to first line anti-microbial defense [171]. Hence, unconventional T cells share two features with innate immune cells: (1) recognition of conserved bacterial compounds, in the context of invariant presenting molecules; and (2) early effector functions.

The TCRs of γδ T cells are composed of γ and δ chains. They are the first T cell subset developing in the fetal thymus. The proportion of the T cell pool comprised of γδ T cells, as well as its composition, vary between mammalian species. In human adults between 0.5% and 16% of circulating T cells are γδ T cells with similar frequencies in the mucosal tissues and peripheral lymphoid organs. In mice, 1–4% of the T cells populating the lymphoid organs express γδ TCRs, but considerably higher proportions are found in the mucosal barrier tissues. 20%–30% of all T cells in the peripheral blood are γδ T cells in cattle and sheep. A subpopulation of human γδ T cells, which is characterized by the use of TCR-Vγ9 and -Vδ2 elements, recognizes bacterial pyrophosphates—(E)-4-hydroxy-3-methyl-but-2-enyl pyrophosphate and isopentenyl pyrophosphate—after their ligation to an innate sensor molecule, butyrophilin 3 (BTN3, CD277). Vγ9/Vδ2 T cells react to Gram-positive as well as Gram-negative bacteria [171]. Some remarkable features have been discovered in γδ T cells: so-called Tγδ17 cells respond with IL-17 secretion upon the primary encounter with antigen without the need for prior clonal expansion. Besides IL-17, the effector functions of γδ T cells include the release of IFN-γ, IL-4 as well as cytolytic activity [32].

**Invariant αβ T cells** use a restricted repertoire of TCR α-chains; the sequences are near germline being modified with very few N nucleotides. These invariant αβ T cells are highly conserved between individuals and recognize non-protein ligands complexed with conserved MHC-Ib molecules [172]. They lie at the interface between innate and adaptive immunity and participate in immune defense against bacteria [171,173,174]. **(Invariant) natural killer T cells (iNKT)** are characterized by TCR α-chains composed of Vα24 (or the murine homolog Vα14) and Jα18. They recognize bacterial or host glycolipids in the context of the conserved MHC-I-like surface molecule CD1d. Upon sensing microbes, which may occur directly by TCR-mediated recognition of bacterial lipid/CD1d complexes or indirectly in response to host lipid/CD1d complexes in combination with cytokines released by activated dendritic cells, iNKT cells can produce large amounts of IFN-γ, IL-4 or IL-17 [175,176]. **Mucosa-associated invariant T cells (MAIT)** are CD4^+^ or CD8^+^ αβ T cells using the TCRα-chain elements Vα7.2 and Jα33 mostly in combination with Vβ2 or Vβ13 TCRβ-chains (human gene elements TRAV1-2, TRAJ33, TRBV20-1 or TRBV6). They recognize bacterial compounds derived from the vitamin B2 metabolic pathway in the context of the non-polymorphic MHC-like molecule M1, which is expressed on the surface of B cells. As their name suggests, MAIT cells accumulate in the mucosal tissues but also in the liver [171,177]. They have been shown to respond to dendritic cells infected with numerous bacterial species including *S. aureus* [177,178]. Similar to MAIT cells, **germline-encoded mycolyl-reactive T cells (GEM)** also use the TCRα-chain element Vα7.2 (TRAV1-2) but recombine it with the joining element TRAJ9. GEM T cells recognize mycolic acids, which are major components of the mycobacterial cell wall, in the context of the invariant MHC-like molecule CD1b [171]. Next generation sequencing revealed the existence of additional invariant T cell subsets using Vα7.2 (TRAV1-2) though the function of these cells has not yet been explored [172].

**Table A2:** Parallels in differentiation and function of innate lymphoid cells (ILCs) and T cells.

Innate Lymphoid Cells				T Cells			
	Inducing Cytokines	Lineage-Specific Transcription Factor	Secreted Cytokines		Inducing Cytokines	Lineage-Specific Transcription Factor	Secreted Cytokines
**ILC1**	IL-12, IL-15, IL-18	Tbet	IFN-γ TNF-α	**T_H_1**	IL-12 IFN-γ	Tbet	IFN-γ IL-2
**ILC2**	IL-25, IL-33, TSLP	Gata3, RORα	IL-4, IL-5, IL-13, amphiregulin	**T_H_2**	IL-4	Gata3	IL-4, IL-5, IL-9, IL-13
**ILC3**	IL-1β, IL-23	RORγt, Ahr	IL-17, IL-22, LT, GM-CSF	**T_H_17**	TGF-β IL1-β, IL-6, IL-21, IL-23	RORγt	IL-17 IL-6
**NK**	IL-12, IL-15, IL-18	Eomes	IFN-γ	**CTL**			IL-2, IFN-γ

Ahr: Arylhydrocarbon receptor; CTL: Cytotoxic T lymphocyte; ILC: innate lymphoid cell; NK: natural killer cell; T_H_: T helper cell.

#### A1.3. Innate Lymphoid Cells, Not T Cells But Similar?

Innate lymphoid cells (ILCs), including NK cells, develop from common lymphoid progenitor cells as do T and B cells. However, unlike the cells of the adaptive immune system, ILCs lack clonotypic T cell or B cell receptors, and they do not undergo clonal selection and expansion when activated. Apart from this, the ILC types, which have been discovered in the last decade, mirror T cell subpopulations in many aspects of their differentiation and function [173]. Table A2 provides an overview of ILCs and their T cell counterparts. ILCs are activated by cytokines in their micro-milieu rather than by antigen recognition. Most ILCs reside in the barrier organs, the skin and mucosal tissues, where they help maintain homeostasis and support tissue repair. In addition, they react very rapidly to infection and cell damage by producing effector cytokines. These can act as signal 3 on T cells directing their differentiation and shaping the quality of the T cell response. Moreover, ILC3s express MHC-II molecules enabling them to present antigen to T cells [179]. Excessive numbers and activity of ILCs have been observed in chronic inflammatory conditions such as Crohn’s disease (ILC1), atopic dermatitis and asthma (ILC2) as well as in psoriasis (ILC3) [173,180].

## References

[1] Fleischer B., Schrezenmeier H. (1988). T-cell stimulation by staphylococcal enterotoxins—Clonally variable response and requirement for major histocompatibility complex class-II molecules on accessory or target-cells. J. Exp. Med..

[2] Grumann D., Nübel U., Bröker B.M. (2014). *Staphylococcus aureus* toxins-their functions and genetics. Infect. Genet. Evol..

[3] Lowy F. (1998). *Staphylococcus aureus* infections. N. Engl. J. Med..

[4] Tong S.Y., Davis J.S., Eichenberger E., Holland T.L., Fowler V.G. (2015). *Staphylococcus aureus* infections: Epidemiology, pathophysiology, clinical manifestations, and management. Clin. Microbiol. Rev..

[5] Wertheim H.F.L., Melles D.C., Vos M.C., van Leeuwen W., van Belkum A., Verbrugh H.A., Nouwen J.I. (2005). The role of nasal carriage in *Staphylococcus aureus* infections. Lancet Infect. Dis..

[6] van Belkum A., Verkaik N.J., de Vogel C.P., Boelens H.A., Verveer J., Nouwen J.L., Verbrugh H.A., Wertheim H.F. (2009). Reclassification of *Staphylococcus aureus* nasal carriage types. J. Infect. Dis..

[7] Brown A.F., Leech J.M., Rogers T.R., McLoughlin R.M. (2014). Staphylococcus aureus Colonization: Modulation of Host Immune Response and Impact on Human Vaccine Design. Front. Immunol..

[8] Laupland K.B., Lyytikainen O., Sogaard M., Kennedy K.J., Knudsen J.D., Ostergaard C., Galbraith J.C., Valiquette L., Jacobsson G., Collignon P. (2013). The changing epidemiology of *Staphylococcus aureus* bloodstream infection: A multinational population-based surveillance study. Clin. Microbiol. Infect..

[9] van Belkum A. (2006). Staphylococcal colonization and infection: Homeostasis *versus* disbalance of human (innate) immunity and bacterial virulence. Curr. Opin. Infect. Dis..

[10] Foster T.J. (2005). Immune evasion by staphylococci. Nature Rev. Microbiol..

[11] Thammavongsa V., Kim H.K., Missiakas D., Schneewind O. (2015). Staphylococcal manipulation of host immune responses. Nat. Rev. Microbiol..

[12] Foster T.J., Geoghegan J.A., Ganesh V.K., Hook M. (2014). Adhesion, invasion and evasion: The many functions of the surface proteins of *Staphylococcus aureus*. Nat. Rev. Microbiol..

[13] Spaan A.N., Surewaard B.G., Nijland R., van Strijp J.A. (2013). Neutrophils *versus*
*Staphylococcus aureus*: A biological tug of war. Annu. Rev. Microbiol..

[14] Laarman A., Milder F., van Strijp J., Rooijakkers S. (2010). Complement inhibition by gram-positive pathogens: Molecular mechanisms and therapeutic implications. J. Mol. Med. (Berl.).

[15] van Kessel K.P., Bestebroer J., van Strijp J.A. (2014). Neutrophil-mediated phagocytosis of *Staphylococcus aureus*. Front. Immunol..

[16] Paul W.E., Paul W.E. (2013). The immune system. Fundamental Immunology.

[17] McHeyzer-Williams M., Okitsu S., Wang N., McHeyzer-Williams L. (2012). Molecular programming of B cell memory. Nat. Rev. Immunol..

[18] O´Shea J., Paul W.E. (2013). Helper T-cell differentiation and plasticity. Fundamental Immunology.

[19] Lowy F.D. (2000). Is *Staphylococcus aureus* an intracellular pathogen?. Trends Microbiol..

[20] Lieberman J., Paul W.E. (2013). Cell-mediated cytotoxicity. Fundamental Immunology.

[21] Spellberg B., Daum R. (2012). Development of a vaccine against *Staphylococcus aureus*. Semin. Immunopathol..

[22] Proctor R.A. (2012). Is there a future for a *Staphylococcus aureus* vaccine?. Vaccine.

[23] von Köckritz-Blickwede M., Rohde M., Oehmcke S., Miller L.S., Cheung A.L., Herwald H., Foster S., Medina E. (2008). Immunological mechanisms underlying the genetic predisposition to severe *Staphylococcus aureus* infection in the mouse model. Am. J. Pathol..

[24] Nippe N., Varga G., Holzinger D., Löffler B., Medina E., Becker K., Roth J., Ehrchen J.M., Sunderkötter C. (2011). Subcutaneous infection with *S. aureus* in mice reveals association of resistance with influx of neutrophils and Th2 response. J. Invest. Dermatol..

[25] Montgomery C.P., Daniels M., Zhao F., Alegre M.L., Chong A.S., Daum R.S. (2014). Protective immunity against recurrent *Staphylococcus aureus* skin infection requires antibody and interleukin-17A. Infect. Immun..

[26] Ziegler C., Goldmann O., Hobeika E., Geffers R., Peters G., Medina E. (2011). The dynamics of T cells during persistent *Staphylococcus aureus* infection: From antigen-reactivity to *in vivo* anergy. EMBO Mol. Med..

[27] Tebartz C., Horst S.A., Sparwasser T., Huehn J., Beineke A., Peters G., Medina E. (2015). A major role for myeloid-derived suppressor cells and a minor role for regulatory T cells in immunosuppression during *Staphylococcus aureus* infection. J. Immunol..

[28] Archer N.K., Harro J.M., Shirtliff M.E. (2013). Clearance of *Staphylococcus aureus* nasal carriage is T cell dependent and mediated through interleukin-17A expression and neutrophil influx. Infect. Immun..

[29] Dong C. (2008). TH17 cells in development: An updated view of their molecular identity and genetic programming. Nat. Rev. Immunol..

[30] Shibata K., Yamada H., Hara H., Kishihara K., Yoshikai Y. (2007). Resident Vdelta1+ gammadelta T cells control early infiltration of neutrophils after *Escherichia coli* infection via IL-17 production. J. Immunol..

[31] Happel K.I., Zheng M., Young E., Quinton L.J., Lockhart E., Ramsay A.J., Shellito J.E., Schurr J.R., Bagby G.J., Nelson S. (2003). Cutting edge: Roles of Toll-like receptor 4 and IL-23 in IL-17 expression in response to *Klebsiella pneumoniae* infection. J. Immunol..

[32] Chien Y.H., Zeng X., Prinz I. (2013). The natural and the inducible: Interleukin (IL)-17-producing gammadelta T cells. Trends Immunol..

[33] Ishigame H., Kakuta S., Nagai T., Kadoki M., Nambu A., Komiyama Y., Fujikado N., Tanahashi Y., Akitsu A., Kotaki H. (2009). Differential roles of interleukin-17A and -17F in host defense against mucoepithelial bacterial infection and allergic responses. Immunity.

[34] Cho J.S., Pietras E.M., Garcia N.C., Ramos R.I., Farzam D.M., Monroe H.R., Magorien J.E., Blauvelt A., Kolls J.K., Cheung A.L. (2010). IL-17 is essential for host defense against cutaneous *Staphylococcus aureus* infection in mice. J. Clin. Invest..

[35] Maher B.M., Mulcahy M.E., Murphy A.G., Wilk M., O’Keeffe K.M., Geoghegan J.A., Lavelle E.C., McLoughlin R.M. (2013). Nlrp-3-driven interleukin 17 production by γδT cells controls infection outcomes during *Staphylococcus aureus* surgical site infection. Infect. Immun..

[36] Molne L., Corthay A., Holmdahl R., Tarkowski A. (2003). Role of gamma/delta T cell receptor-expressing lymphocytes in cutaneous infection caused by *Staphylococcus aureus*. Clin. Exp. Immunol..

[37] Murphy A.G., O’Keeffe K.M., Lalor S.J., Maher B.M., Mills K.H., McLoughlin R.M. (2015). Correction: *Staphylococcus aureus* Infection of mice expands a population of memory γδ T cells that are protective against subsequent infection. J. Immunol..

[38] Lin L., Ibrahim A.S., Xu X., Farber J.M., Avanesian V., Baquir B., Fu Y., French S.W., Edwards J.E., Spellberg B. (2009). Th1-Th17 cells mediate protective adaptive immunity against *Staphylococcus aureus* and *Candida albicans* infection in mice. PLoS Pathog..

[39] Narita K., Hu D.L., Mori F., Wakabayashi K., Iwakura Y., Nakane A. (2010). Role of interleukin-17A in cell-mediated protection against *Staphylococcus aureus* infection in mice immunized with the fibrinogen-binding domain of clumping factor A. Infect. Immun..

[40] Wang L., Kamath A., Das H., Li L., Bukowski J.F. (2001). Antibacterial effect of human V gamma 2V delta 2 T cells *in vivo*. J. Clin. Invest..

[41] Parker D., Ryan C.L., Alonzo F., Torres V.J., Planet P.J., Prince A.S. (2015). CD4+ T cells promote the pathogenesis of *Staphylococcus aureus* pneumonia. J. Infect. Dis..

[42] Cheng P., Liu T., Zhou W.Y., Zhuang Y., Peng L.S., Zhang J.Y., Yin Z.N., Mao X.H., Guo G., Shi Y. (2012). Role of gamma-delta T cells in host response against *Staphylococcus aureus*-induced pneumonia. BMC Immunol..

[43] Kudva A., Scheller E.V., Robinson K.M., Crowe C.R., Choi S.M., Slight S.R., Khader S.A., Dubin P.J., Enelow R.I., Kolls J.K. (2011). Influenza A inhibits Th17-mediated host defense against bacterial pneumonia in mice. J. Immunol..

[44] Ohlsen K., Lorenz U. (2010). Immunotherapeutic strategies to combat staphylococcal infections. Int. J. Med. Microbiol..

[45] Joshi A., Pancari G., Cope L., Bowman E.P., Cua D., Proctor R.A., McNeely T. (2012). Immunization with *Staphylococcus aureus* iron regulated surface determinant B (IsdB) confers protection via Th17/IL17 pathway in a murine sepsis model. Hum. Vaccines Immunother..

[46] Misstear K., McNeela E.A., Murphy A.G., Geoghegan J.A., O’Keeffe K.M., Fox J., Chan K., Heuking S., Collin N., Foster T.J. (2014). Targeted nasal vaccination provides antibody-independent protection against *Staphylococcus aureus*. J. Infect. Dis..

[47] Brown A.F., Murphy A.G., Lalor S.J., Leech J.M., O’Keeffe K.M., Mac Aogain M., O’Halloran D.P., Lacey K.A., Tavakol M., Hearnden C.H. (2015). Memory Th1 cells are protective in invasive *Staphylococcus aureus* infection. PLoS Pathog..

[48] Wang X.Y., Huang Z.X., Chen Y.G., Lu X., Zhu P., Wen K., Fu N., Liu B.Y. (2015). A multiple antigenic peptide mimicking peptidoglycan induced T cell responses to protect mice from systemic infection with *Staphylococcus aureus*. PLoS ONE.

[49] Murphy A.G., O’Keeffe K.M., Lalor S.J., Maher B.M., Mills K.H., McLoughlin R.M. (2014). *Staphylococcus aureus* infection of mice expands a population of memory gd T cells that are protective against subsequent infection. J. Immunol..

[50] Schroeder J.W. (2012). Mastitis control programs: Bovine mastitis and milking management. Document AS-1129.

[51] Barkema H.W., Green M.J., Bradley A.J., Zadoks R.N. (2009). Invited review: The role of contagious disease in udder health. J. Dairy Sci..

[52] Miller G.Y., Bartlett P.C., Lance S.E., Anderson J., Heider L.E. (1993). Costs of clinical mastitis and mastitis prevention in dairy herds. J. Am. Vet. Med. Assoc..

[53] Fabres-Klein M.H., Aguilar A.P., Silva M.P., Silva D.M., Ribon A.O. (2014). Moving towards the immunodiagnosis of staphylococcal intramammary infections. Eur. J. Clin. Microbiol. Infect. Dis..

[54] Seegers H., Fourichon C., Beaudeau F. (2003). Production effects related to mastitis and mastitis economics in dairy cattle herds. Vet. Res..

[55] Soltys J., Quinn M.T. (1999). Selective recruitment of T-cell subsets to the udder during staphylococcal and streptococcal mastitis: Analysis of lymphocyte subsets and adhesion molecule expression. Infect. Immun..

[56] Gronlund U., Johannisson A., Persson Waller K. (2006). Changes in blood and milk lymphocyte sub-populations during acute and chronic phases of *Staphylococcus aureus* induced bovine mastitis. Res. Vet. Sci..

[57] Rivas A.L., Quimby F.W., Coksaygan O., Olmstead L., Lein D.H. (2000). Longitudinal evaluation of CD4+ and CD8+ peripheral blood and mammary gland lymphocytes in cows experimentally inoculated with *Staphylococcus aureus*. Can. J. Vet. Res..

[58] Park Y.H., Joo Y.S., Park J.Y., Moon J.S., Kim S.H., Kwon N.H., Ahn J.S., Davis W.C., Davies C.J. (2004). Characterization of lymphocyte subpopulations and major histocompatibility complex haplotypes of mastitis-resistant and susceptible cows. J. Vet. Sci..

[59] Chang B.S., Bohach G.A., Lee S.U., Davis W.C., Fox L.K., Ferens W.A., Seo K.S., Koo H.C., Kwon N.H., Park Y.H. (2005). Immunosuppression by T regulatory cells in cows infected with *Staphylococcal superantigen*. J. Vet. Sci..

[60] Riollet C., Rainard P., Poutrel B. (2001). Cell subpopulations and cytokine expression in cow milk in response to chronic *Staphylococcus aureus* infection. J. Dairy Sci..

[61] Bharathan M., Mullarky I.K. (2011). Targeting mucosal immunity in the battle to develop a mastitis vaccine. J. Mammary Gland Biol. Neoplasia.

[62] Pereira U.P., Oliveira D.G.S., Mesquita L.R., Costa G.M., Pereira L.J. (2011). Efficacy of *Staphylococcus aureus* vaccines for bovine mastitis: A systematic review. Vet. Microbiol..

[63] Péton V., Le Loir Y. (2014). *Staphylococcus aureus* in veterinary medicine. Infect. Genet. Evol..

[64] Camussone C.M., Pujato N., Renna M.S., Veaute C.M., Morein B., Marcipar I.S., Calvinho L.F. (2014). Immune response and functional role of antibodies raised in heifers against a *Staphylococcus aureus* CP5 lysate and recombinant antigens vaccine formulated with Iscom Matrix adjuvant. Vet. Immunol. Immunopathol..

[65] Schukken Y.H., Bronzo V., Locatelli C., Pollera C., Rota N., Casula A., Testa F., Scaccabarozzi L., March R., Zalduendo D. (2014). Efficacy of vaccination on *Staphylococcus aureus* and coagulase-negative staphylococci intramammary infection dynamics in 2 dairy herds. J. Dairy Sc..

[66] Lee J.-W., O’Brien C.N., Guidry A.J., Paape M.J., Shafer-Weaver K.A., Zhao X. (2005). Effect of a trivalent vaccine against *Staphylococcus aureus* mastitis lymphocyte subpopulations, antibody production, and neutrophil phagocytosis. Can. J. Vet. Res..

[67] Prenafeta A., March R., Foix A., Casals I., Costa L. (2010). Study of the humoral immunological response after vaccination with a *Staphylococcus aureus* biofilm-embedded bacterin in dairy cows: Possible role of the exopolysaccharide specific antibody production in the protection from *Staphylococcus aureus* induced mastitis. Vet. Immunol. Immunopathol..

[68] Watson D., McCOLL M., Davies H. (1996). Field trial of a staphylococcal mastitis vaccine in dairy herds: Clinical, subclinical and microbiological assessments. Aust. Vet. J..

[69] Middleton J.R., Ma J., Rinehart C.L., Taylor V.N., Luby C.D., Steevens B.J. (2006). Efficacy of different Lysigin formulations in the prevention of *Staphylococcus aureus* intramammary infection in dairy heifers. J. Dairy Res..

[70] Middleton J.R. (2008). *Staphylococcus aureus* antigens and challenges in vaccine development. Expert Rev. Vaccines.

[71] Middleton J.R., Luby C.D., Adams D.S. (2009). Efficacy of vaccination against staphylococcal mastitis: A review and new data. Vet. Microbiol..

[72] Pankey J.W., Boddie N.T., Watts J.L., Nickerson S.C. (1985). Evaluation of Protein A and a commercial bacterin as vaccines against *Staphylococcus aureus* mastitis by experimental challenge. J. Dairy Sci..

[73] Buzzola F.R., Alvarez L.P., Tuchscherr L.P.N., Barbagelata M.S., Lattar S.M., Calvinho L., Sordelli D.O. (2007). Differential abilities of capsulated and noncapsulated *Staphylococcus aureus* isolates from diverse *agr* groups to invade mammary epithelial cells. Infect. Immun..

[74] Pellegrino M., Giraudo J., Raspanti C., Nagel R., Odierno L., Primo V., Bogni C. (2008). Experimental trial in heifers vaccinated with *Staphylococcus aureus* avirulent mutant against bovine mastitis. Vet. Microbiol..

[75] Pellegrino M., Giraudo J., Raspanti C., Odierno L., Bogni C. (2010). Efficacy of immunization against bovine mastitis using a *Staphylococcus aureus* avirulent mutant vaccine. Vaccine.

[76] Lawrence P.K., Rokbi B., Arnaud-Barbe N., Sutten E.L., Norimine J., Lahmers K.K., Brown W.C. (2012). CD4 T cell antigens from *Staphylococcus aureus* Newman strain identified following immunization with heat-killed bacteria. Clin. Vaccine Immunol..

[77] Pujol J., Bouillenne F., Farnir F., Dufrasne I., Mainil J., Galleni M., Lekeux P., Bureau F., Fievez L. (2015). Generation of a soluble recombinant trimeric form of bovine CD40L and its potential use as a vaccine adjuvant in cows. Vet. Immunol. Immunopathol..

[78] van Belkum A., Melles D.C., Nouwen J., van Leeuwen W.B., van Wamel W., Vos M.C., Wertheim H.F., Verbrugh H.A. (2009). Co-evolutionary aspects of human colonisation and infection by *Staphylococcus aureus*. Infect. Genet. Evol..

[79] Chandesris M.O., Melki I., Natividad A., Puel A., Fieschi C., Yun L., Thumerelle C., Oksenhendler E., Boutboul D., Thomas C. (2012). Autosomal dominant STAT3 deficiency and hyper-IgE syndrome: Molecular, cellular, and clinical features from a French national survey. Medicine (Baltimore).

[80] Cook M.C., Tangye S.G. (2009). Primary immune deficiencies affecting lymphocyte differentiation: Lessons from the spectrum of resulting infections. Int. Immunol..

[81] Milner J.D., Brenchley J.M., Laurence A., Freeman A.F., Hill B.J., Elias K.M., Kanno Y., Spalding C., Elloumi H.Z., Paulson M.L. (2008). Impaired T(H)17 cell differentiation in subjects with autosomal dominant hyper-IgE syndrome. Nature.

[82] Ma C.S., Chew G.Y., Simpson N., Priyadarshi A., Wong M., Grimbacher B., Fulcher D.A., Tangye S.G., Cook M.C. (2008). Deficiency of Th17 cells in hyper IgE syndrome due to mutations in STAT3. J. Exp. Med..

[83] Renner E.D., Rylaarsdam S., Anover-Sombke S., Rack A.L., Reichenbach J., Carey J.C., Zhu Q., Jansson A.F., Barboza J., Schimke L.F. (2008). Novel signal transducer and activator of transcription 3 (STAT3) mutations, reduced T(H)17 cell numbers, and variably defective STAT3 phosphorylation in hyper-IgE syndrome. J. Allergy Clin. Immunol..

[84] Holland S.M., DeLeo F.R., Elloumi H.Z., Hsu A.P., Uzel G., Brodsky N., Freeman A.F., Demidowich A., Davis J., Turner M.L. (2007). STAT3 mutations in the hyper-IgE syndrome. N. Engl. J. Med..

[85] Craven D.E. (1995). *Staphylococcus aureus* colonisation and bacteraemia in persons infected with human immunodeficiency virus: A dynamic interaction with the host. J. Chemother..

[86] Reddy E.A., Shaw A.V., Crump J.A. (2010). Community-acquired bloodstream infections in Africa: A systematic review and meta-analysis. Lancet Infect. Dis..

[87] Laupland K.B., Ross T., Gregson D.B. (2008). *Staphylococcus aureus* bloodstream infections: Risk factors, outcomes, and the influence of methicillin resistance in Calgary, Canada, 2000–2006. J. Infect. Dis..

[88] Wiese L., Mejer N., Schonheyder H.C., Westh H., Jensen A.G., Larsen A.R., Skov R., Benfield T., Danish Staphylococcal Bacteraemia Study Group (2013). A nationwide study of comorbidity and risk of reinfection after *Staphylococcus aureus* bacteraemia. J. Infect..

[89] Bröker B.M., Holtfreter S., Bekeredjian-Ding I. (2014). Immune control of *Staphylocuccus aureus*—Regulation and counter-regulation of the adaptive immune response. Int. J. Med. Microbiol..

[90] Bröker B.M., van Belkum A. (2011). Immune proteomics of *Staphylococcus aureus*. Proteomics.

[91] Vytvytska O., Nagy E., Bluggel M., Meyer H., Kurzbauer R., Huber L., Klade C. (2002). Identification of vaccine candidate antigens of *Staphylococcus aureus* by serological proteome analysis. Proteomics.

[92] Kolata J., Bode L.G., Holtfreter S., Steil L., Kusch H., Holtfreter B., Albrecht D., Hecker M., Engelmann S., van Belkum A. (2011). Distinctive patterns in the human antibody response to *Staphylococcus aureus* bacteremia in carriers and non-carriers. Proteomics.

[93] Holtfreter S., Roschack K., Eichler P., Eske K., Holtfreter B., Kohler C., Engelmann S., Hecker M., Greinacher A., Bröker B.M. (2006). *Staphylococcus aureus* carriers neutralize superantigens by antibodies specific for their colonizing strain: A potential explanation for their improved prognosis in severe sepsis. J. Infect. Dis..

[94] Crotty S. (2015). A brief history of T cell help to B cells. Nat. Rev. Immunol..

[95] Crotty S. (2011). Follicular helper CD4 T cells (TFH). Annu. Rev. Immunol..

[96] Zielinski C.E., Corti D., Mele F., Pinto D., Lanzavecchia A., Sallusto F. (2011). Dissecting the human immunologic memory for pathogens. Immunol. Rev..

[97] Kolata J., Kühbandner I., Link C., Normann N., Weidenmaier C., Bröker B. (2015). The fall of a dogma? Unexpectedly high T cell memory response to *Staphylococcus aureus* in humans. J. Infect. Dis..

[98] Zielinski C.E., Mele F., Aschenbrenner D., Jarrossay D., Ronchi F., Gattorno M., Monticelli S., Lanzavecchia A., Sallusto F. (2012). Pathogen-induced human TH17 cells produce IFN-gamma or IL-10 and are regulated by IL-1beta. Nature.

[99] Stentzel S., Teufelberger A., Nordengrün M., Kolata J., Schmidt F., van Crombruggen K., Michalik S., Kumpfmüller J., Tischer S., Schweder T. (2016). Spls are pacemakers of allergic airway reactions to *Staphylococcus aureus*.

[100] Frodermann V., Chau T.A., Sayedyahossein S., Toth J.M., Heinrichs D.E., Madrenas J. (2011). A modulatory interleukin-10 response to staphylococcal peptidoglycan prevents Th1/Th17 adaptive immunity to *Staphylococcus aureus*. J. Infect. Dis..

[101] Belkaid Y., Tarbell K. (2009). Regulatory T cells in the control of host-microorganism interactions (*). Annu. Rev. Immunol..

[102] Belkaid Y., Rouse B.T. (2005). Natural regulatory T cells in infectious disease. Nat. Immunol..

[103] Fowler V.G., Allen K.B., Moreira E.D., Moustafa M., Isgro F., Boucher H.W., Corey G.R., Carmeli Y., Betts R., Hartzel J.S. (2013). Effect of an investigational vaccine for preventing *Staphylococcus aureus* infections after cardiothoracic surgery: A randomized trial. JAMA.

[104] Niebuhr M., Gathmann M., Scharonow H., Mamerow D., Mommert S., Balaji H., Werfel T. (2011). Staphylococcal alpha-toxin is a strong inducer of interleukin-17 in humans. Infect. Immun..

[105] Breuer K., Wittmann M., Kempe K., Kapp A., Mai U., Dittrich-Breiholz O., Kracht M., Mrabet-Dahbi S., Werfel T. (2005). Alpha-toxin is produced by skin colonizing *Staphylococcus aureus* and induces a T helper type 1 response in atopic dermatitis. Clin. Exp. Allergy.

[106] Warmerdam P.A., Vanderlick K., Vandervoort P., De Smedt H., Plaisance S., De Maeyer M., Collen D. (2002). Staphylokinase-specific cell-mediated immunity in humans. J. Immunol..

[107] Weidenmaier C., McLoughlin R.M., Lee J.C. (2010). The zwitterionic cell wall teichoic acid of *Staphylococcus aureus* provokes skin abscesses in mice by a novel CD4+ T-cell-dependent mechanism. PLoS ONE.

[108] Levy J., Licini L., Haelterman E., Moris P., Lestrate P., Damaso S., Van Belle P., Boutriau D. (2015). Safety and immunogenicity of an investigational 4-component *Staphylococcus aureus* vaccine with or without AS03B adjuvant: Results of a randomized phase I trial. Hum. Vaccines Immunother..

[109] Nissen M., Marshall H., Richmond P., Shakib S., Jiang Q., Cooper D., Rill D., Baber J., Eiden J., Gruber W. (2015). A randomized phase I study of the safety and immunogenicity of three ascending dose levels of a 3-antigen *Staphylococcus aureus* vaccine (SA3Ag) in healthy adults. Vaccine.

[110] Projan S.J., Nesin M., Dunman P.M. (2006). Staphylococcal vaccines and immunotherapy: To dream the impossible dream?. Curr. Opin. Pharmacol..

[111] Verkaik N.J., van Wamel W.J., van Belkum A. (2011). Immunotherapeutic approaches against *Staphylococcus aureus*. Immunotherapy.

[112] Fowler V.G., Proctor R.A. (2014). Where does a *Staphylococcus aureus* vaccine stand?. Clin. Microbiol. Infect..

[113] Bagnoli F., Bertholet S., Grandi G. (2012). Inferring reasons for the failure of *Staphylococcus aureus* vaccines in clinical trials. Front. Cell. Infect. Microbiol..

[114] Fattom A., Matalon A., Buerkert J., Taylor K., Damaso S., Boutriau D. (2015). Efficacy profile of a bivalent *Staphylococcus aureus* glycoconjugated vaccine in adults on hemodialysis: Phase III randomized study. Hum. Vaccines Immunother..

[115] Jansen K.U., Girgenti D.Q., Scully I.L., Anderson A.S. (2013). Vaccine review: “*Staphyloccocus aureus* vaccines: Problems and prospects”. Vaccine.

[116] McNeely T.B., Shah N.A., Fridman A., Joshi A., Hartzel J.S., Keshari R.S., Lupu F., DiNubile M.J. (2014). Mortality among recipients of the Merck V710 *Staphylococcus aureus* vaccine after postoperative *S. aureus* infections: An analysis of possible contributing host factors. Hum. Vaccines Immunother..

[117] Löffler B., Tuchscherr L., Niemann S., Peters G. (2014). *Staphylococcus aureus* persistence in non-professional phagocytes. Int. J. Med. Microbiol..

[118] Grosz M., Kolter J., Paprotka K., Winkler A.C., Schafer D., Chatterjee S.S., Geiger T., Wolz C., Ohlsen K., Otto M. (2014). Cytoplasmic replication of *Staphylococcus aureus* upon phagosomal escape triggered by phenol-soluble modulin alpha. Cell. Microbiol..

[119] Thwaites G.E., Gant V. (2011). Are bloodstream leukocytes Trojan Horses for the metastasis of *Staphylococcus aureus*?. Nat. Rev. Microbiol..

[120] Tuchscherr L., Medina E., Hussain M., Volker W., Heitmann V., Niemann S., Holzinger D., Roth J., Proctor R.A., Becker K. (2011). *Staphylococcus aureus* phenotype switching: An effective bacterial strategy to escape host immune response and establish a chronic infection. EMBO Mol. Med..

[121] Tuchscherr L., Heitmann V., Hussain M., Viemann D., Roth J., von Eiff C., Peters G., Becker K., Loffler B. (2010). *Staphylococcus aureus* small-colony variants are adapted phenotypes for intracellular persistence. J. Infect. Dis..

[122] Surmann K., Simon M., Hildebrandt P., Pförtner H., Michalik S., Stentzel S., Steil L., Dhople V.M., Bernhardt J., Schlüter R. (2015). A proteomic perspective of the interplay of *Staphylococcus aureus* and human alveolar epithelial cells during infection. J. Proteomics.

[123] Surmann K., Michalik S., Hildebrandt P., Gierok P., Depke M., Brinkmann L., Bernhardt J., Salazar M.G., Sun Z., Shteynberg D. (2014). Comparative proteome analysis reveals conserved and specific adaptation patterns of *Staphylococcus aureus* after internalization by different types of human non-professional phagocytic host cells. Front. Microbiol..

[124] Holzinger D., Gieldon L., Mysore V., Nippe N., Taxman D.J., Duncan J.A., Broglie P.M., Marketon K., Austermann J., Vogl T. (2012). *Staphylococcus aureus* Panton-Valentine leukocidin induces an inflammatory response in human phagocytes via the NLRP3 inflammasome. J. Leukoc. Biol..

[125] Kubica M., Guzik K., Koziel J., Zarebski M., Richter W., Gajkowska B., Golda A., Maciag-Gudowska A., Brix K., Shaw L. (2008). A potential new pathway for *Staphylococcus aureus* dissemination: The silent survival of *S. aureus* phagocytosed by human monocyte-derived macrophages. PLoS ONE.

[126] Smith R.P., Baltch A.L., Ritz W.J., Michelsen P.B., Bopp L.H. (2010). IFN-gamma enhances killing of methicillin-resistant *Staphylococcus aureus* by human monocytes more effectively than GM-CSF in the presence of daptomycin and other antibiotics. Cytokine.

[127] Penaloza-MacMaster P., Barber D.L., Wherry E.J., Provine N.M., Teigler J.E., Parenteau L., Blackmore S., Borducchi E.N., Larocca R.A., Yates K.B. (2015). Vaccine-elicited CD4 T cells induce immunopathology after chronic LCMV infection. Science.

[128] McLoughlin R.M., Lee J.C., Kasper D.L., Tzianabos A.O. (2008). IFN-gamma regulated chemokine production determines the outcome of *Staphylococcus aureus* infection. J. Immunol..

[129] Barnes P.J. (2009). Intrinsic asthma: Not so different from allergic asthma but driven by superantigens?. Clin. Exp. Allergy.

[130] Bachert C., Zhang N. (2012). Chronic rhinosinusitis and asthma: Novel understanding of the role of IgE ‘above atopy’. J. Intern. Med..

[131] Davis M.F., Peng R.D., McCormack M.C., Matsui E.C. (2015). *Staphylococcus aureus* colonization is associated with wheeze and asthma among US children and young adults. J. Allergy Clin. Immunol..

[132] Wills-Karp M., Lewkowich I.P., Paul W.E. (2013). Immunologic mechanism of allergic disorders. Fundamental Immunology.

[133] Berube B.J., Bubeck Wardenburg J. (2013). *Staphylococcus aureus* alpha-toxin: Nearly a century of intrigue. Toxins.

[134] Inoshima I., Inoshima N., Wilke G.A., Powers M.E., Frank K.M., Wang Y., Wardenburg J.B. (2011). A *Staphylococcus aureus* pore-forming toxin subverts the activity of ADAM10 to cause lethal infection in mice. Nat. Med..

[135] Brauweiler A.M., Bin L., Kim B.E., Oyoshi M.K., Geha R.S., Goleva E., Leung D.Y. (2012). Filaggrin-dependent secretion of sphingomyelinase protects against staphylococcal alpha-toxin-induced keratinocyte death. J. Allergy Clin. Immunol..

[136] Nakamura Y., Oscherwitz J., Cease K.B., Chan S.M., Munoz-Planillo R., Hasegawa M., Villaruz A.E., Cheung G.Y.C., McGavin M.J., Travers J.B. (2013). Staphylococcus δ-toxin induces allergic skin disease by activating mast cells. Nature.

[137] Huvenne W., Hellings P.W., Bachert C. (2013). Role of staphylococcal superantigens in airway disease. Int. Arch. Allergy Immunol..

[138] Bachert C., Gevaert P., van Cauwenberge P. (2002). *Staphylococcus aureus* enterotoxins: A key in airway disease?. Allergy.

[139] Wilke G.A., Bubeck Wardenburg J. (2010). Role of a disintegrin and metalloprotease 10 in *Staphylococcus aureus* alpha-hemolysin-mediated cellular injury. Proc. Natl. Acad. Sci. USA.

[140] Alonzo F., Kozhaya L., Rawlings S.A., Reyes-Robles T., DuMont A.L., Myszka D.G., Landau N.R., Unutmaz D., Torres V.J. (2013). CCR5 is a receptor for *Staphylococcus aureus* leukotoxin ED. Nature.

[141] Marrack P., Kappler J. (1990). The staphylococcal enterotoxins and their relatives. Science.

[142] Spaulding A.R., Salgado-Pabon W., Kohler P.L., Horswill A.R., Leung D.Y., Schlievert P.M. (2013). Staphylococcal and streptococcal superantigen exotoxins. Clin. Microbiol. Rev..

[143] Xu S.X., McCormick J.K. (2012). Staphylococcal superantigens in colonization and disease. Front. Cell. Infect. Microbiol..

[144] Hu D.L., Zhu G., Mori F., Omoe K., Okada M., Wakabayashi K., Kaneko S., Shinagawa K., Nakane A. (2007). Staphylococcal enterotoxin induces emesis through increasing serotonin release in intestine and it is downregulated by cannabinoid receptor 1. Cell. Microbiol..

[145] Holtfreter S., Grumann D., Schmudde M., Nguyen H.T., Eichler P., Strommenger B., Kopron K., Kolata J., Giedrys-Kalemba S., Steinmetz I. (2007). Clonal distribution of superantigen genes in clinical *Staphylococcus aureus* isolates. J. Clin. Microbiol..

[146] Baba T., Takeuchi F., Kuroda M., Yuzawa H., Aoki K., Oguchi A., Nagai Y., Iwama N., Asano K., Naimi T. (2002). Genome and virulence determinants of high virulence community-acquired MRSA. Lancet.

[147] Becker K., Friedrich A.W., Lubritz G., Weilert M., Peters G., von Eiff C. (2003). Prevalence of genes encoding pyrogenic toxin superantigens and exfoliative toxins among strains of *Staphylococcus aureus* isolated from blood and nasal specimens. J. Clin. Microbiol..

[148] Holtfreter S., Bauer K., Thomas D., Feig C., Lorenz V., Roschack K., Friebe E., Selleng K., Lövenich S., Greve T. (2004). *egc*-Encoded superantigens from *Staphylococcus aureus* are neutralized by human sera much less efficiently than are classical staphylococcal enterotoxins or toxic shock syndrome toxin. Infect. Immun..

[149] Jarraud S., Peyrat M.A., Lim A., Tristan A., Bes M., Mougel C., Etienne J., Vandenesch F., Bonneville M., Lina G. (2001). egc, a highly prevalent operon of enterotoxin gene, forms a putative nursery of superantigens in *Staphylococcus aureus*. J. Immunol..

[150] Grumann D., Scharf S.S., Holtfreter S., Kohler C., Steil L., Engelmann S., Hecker M., Völker U., Bröker B.M. (2008). Immune cell activation by enterotoxin gene cluster (*egc*)-encoded and non-*egc* superantigens from *Staphylococcus aureus*. J. Immunol..

[151] Fraser J.D., Proft T. (2008). The bacterial superantigen and superantigen-like proteins. Immunol Rev.

[152] Proft T., Fraser J. (2003). Bacterial Superantigens. Clin. Exp. Immunol..

[153] Fraser J., Arcus V., Kong P., Baker E., Proft T. (2000). Superantigens—powerful modifiers of the immune system. Mol Med Today.

[154] Fast D.J., Schlievert P.M., Nelson R.D. (1988). Nonpurulent response to toxic shock syndrome toxin 1-producing *Staphylococcus aureus*. Relationship to toxin-stimulated production of tumor necrosis factor. J. Immunol..

[155] DeVries A.S., Lesher L., Schlievert P.M., Rogers T., Villaume L.G., Danila R., Lynfield R. (2011). Staphylococcal toxic shock syndrome 2000–2006: Epidemiology, clinical features, and molecular characteristics. PLoS ONE.

[156] Grumann D., Ruotsalainen E., Kolata J., Kuusela P., Jarvinen A., Kontinen V.P., Bröker B.M., Holtfreter S. (2011). Characterization of infecting strains and superantigen-neutralizing antibodies in *Staphylococcus aureus* bacteremia. Clin. Vaccine Immunol..

[157] Xu S.X., Kasper K.J., Zeppa J.J., McCormick J.K. (2015). Superantigens modulate bacterial density during *Staphylococcus aureus* nasal colonization. Toxins.

[158] Xu S.X., Gilmore K.J., Szabo P.A., Zeppa J.J., Baroja M.L., Haeryfar S.M., McCormick J.K. (2014). Superantigens subvert the neutrophil response to promote abscess formation and enhance *Staphylococcus aureus* survival *in vivo*. Infect. Immun..

[159] Chau T.A., McCully M.L., Brintnell W., An G., Kasper K.J., Vines E.D., Kubes P., Haeryfar S.M., McCormick J.K., Cairns E. (2009). Toll-like receptor 2 ligands on the staphylococcal cell wall downregulate superantigen-induced T cell activation and prevent toxic shock syndrome. Nat. Med..

[160] Schreiner J., Kretschmer D., Klenk J., Otto M., Buhring H.J., Stevanovic S., Wang J.M., Beer-Hammer S., Peschel A., Autenrieth S.E. (2013). *Staphylococcus aureus* phenol-soluble modulin peptides modulate dendritic cell functions and increase *in vitro* priming of regulatory T cells. J. Immunol..

[161] Davies M.M., Chien Y.-H., Paul W.E. (2013). T-lymphocytes. Fundamental Immunology.

[162] Blattman J.N., Antia R., Sourdive D.J., Wang X., Kaech S.M., Murali-Krishna K., Altman J.D., Ahmed R. (2002). Estimating the precursor frequency of naive antigen-specific CD8 T cells. J. Exp. Med..

[163] Geiger R., Duhen T., Lanzavecchia A., Sallusto F. (2009). Human naive and memory CD4+ T cell repertoires specific for naturally processed antigens analyzed using libraries of amplified T cells. J. Exp. Med..

[164] Miller J.D., van der Most R.G., Akondy R.S., Glidewell J.T., Albott S., Masopust D., Murali-Krishna K., Mahar P.L., Edupuganti S., Lalor S. (2008). Human effector and memory CD8+ T cell responses to smallpox and yellow fever vaccines. Immunity.

[165] Trombetta E.S., Mellman I. (2005). Cell biology of antigen processing *in vitro* and *in vivo*. Annu. Rev. Immunol..

[166] Crotty S., Kaech S.M., Schoenberger S.P., Paul W.E. (2013). Immunologic memory. Fundamental Immunology.

[167] Schmitt N., Ueno H. (2015). Regulation of human helper T cell subset differentiation by cytokines. Curr. Opin. Immunol..

[168] Stavnezer J., Guikema J.E., Schrader C.E. (2008). Mechanism and regulation of class switch recombination. Annu. Rev. Immunol..

[169] Kaech S.M., Cui W. (2012). Transcriptional control of effector and memory CD8+ T cell differentiation. Nat. Rev. Immunol..

[170] Hale J.S., Ahmed R. (2015). Memory T follicular helper CD4 T cells. Front. Immunol..

[171] Liuzzi A.R., McLaren J.E., Price D.A., Eberl M. (2015). Early innate responses to pathogens: Pattern recognition by unconventional human T-cells. Curr. Opin. Immunol..

[172] van Schaik B., Klarenbeek P., Doorenspleet M., van Kampen A., Moody D.B., de Vries N., Van Rhijn I. (2014). Discovery of invariant T cells by next-generation sequencing of the human TCR alpha-chain repertoire. J. Immunol..

[173] Eberl G., Colonna M., Di Santo J.P., McKenzie A.N. (2015). Innate lymphoid cells. Innate lymphoid cells: A new paradigm in immunology. Science.

[174] Gao Y., Williams A.P. (2015). Role of Innate T Cells in Anti-Bacterial Immunity. Front. Immunol..

[175] Bendelac A., Savage P.B., Teyton L. (2007). The biology of NKT cells. Annu. Rev. Immunol..

[176] Doisne J.M., Soulard V., Becourt C., Amniai L., Henrot P., Havenar-Daughton C., Blanchet C., Zitvogel L., Ryffel B., Cavaillon J.M. (2011). Cutting edge: Crucial role of IL-1 and IL-23 in the innate IL-17 response of peripheral lymph node NK1.1- invariant NKT cells to bacteria. J. Immunol..

[177] Napier R.J., Adams E.J., Gold M.C., Lewinsohn D.M. (2015). The Role of Mucosal Associated Invariant T Cells in Antimicrobial Immunity. Front. Immunol..

[178] Gold M.C., Cerri S., Smyk-Pearson S., Cansler M.E., Vogt T.M., Delepine J., Winata E., Swarbrick G.M., Chua W.J., Yu Y.Y. (2010). Human mucosal associated invariant T cells detect bacterially infected cells. PLoS Biol.

[179] Hepworth M.R., Monticelli L.A., Fung T.C., Ziegler C.G., Grunberg S., Sinha R., Mantegazza A.R., Ma H.L., Crawford A., Angelosanto J.M. (2013). Innate lymphoid cells regulate CD4+ T-cell responses to intestinal commensal bacteria. Nature.

[180] Hazenberg M.D., Spits H. (2014). Human innate lymphoid cells. Blood.

